# P-617. Estimating Pediatric Breakthrough Disease from Pneumococcal Vaccines in France

**DOI:** 10.1093/ofid/ofae631.815

**Published:** 2025-01-29

**Authors:** Kevin Bakker, Rachel Oidtman, Giulio Meleleo, Priscilla Velentgas, Kenneth Klinker, Natalie Banniettis, Kristen A Feemster, Jessica Weaver

**Affiliations:** Merck & Co., Inc., Rahway, NJ, USA, Philadelphia, Pennsylvania; Merck & Co., Inc., Lansdale, Pennsylvania; Wolfram Research, Inc., Champaign, Illinois; Merck & Co., Inc., Rahway, NJ, USA, Philadelphia, Pennsylvania; Merck & Co, Inc, Kenilworth, NJ; Merck & Co., Inc., Lansdale, Pennsylvania; Merck & Co., Inc., Lansdale, Pennsylvania; Merck & Co., Inc., Lansdale, Pennsylvania

## Abstract

**Background:**

In France, effective pneumococcal conjugate vaccines (PCVs) and the high vaccine coverage rate (VCR) of 95% have led to a substantial decline in pediatric invasive pneumococcal disease (IPD) incidence. PCV13 is currently used in a 2+1 dosing regimen; PCV15 and PCV20 are expected to be introduced soon. Breakthrough IPD (bIPD), defined as fully vaccinated individuals who develop vaccine type IPD, persists for some PCV13 serotypes (STs). As ST-specific immune responses decrease with increased PCV valency, understanding the potential impact of PCV15 and PCV20 on PCV13 STs bIPD is important.

**Figure:**
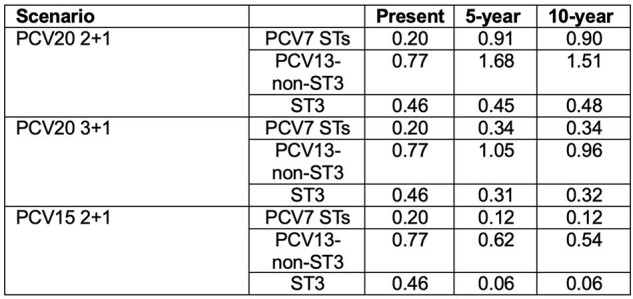

IPD Breakthrough disease incidence per 100,000 children under two years of age.

**Methods:**

We evaluated the expected impact of PCV15 in a 2+1 regimen, or PCV20 in either a 2+1 or 3+1 regimen on PCV13 ST bIPD incidence in children < 2 years of age over 5 and 10 years using a compartmental dynamic transmission model. The model incorporated historical PCV introductions and calibrated age- and ST-specific IPD data spanning 2000-2019. Predicted ST-specific vaccine effectiveness (VE) for PCV15 and PCV20 were used^1,2^. Incidence of bIPD was evaluated across 3 ST groupings: PCV7 STs, PCV13-non-ST3 STs, and ST3. VCRs were maintained at 95% in the model. Results were compared to 2019 IPD incidence.

**Results:**

Using previously published ST-specific VE predictions, routine use of PCV15 in children led to fewer PCV13 ST bIPD cases than use of PCV20. PCV15 reduced bIPD incidence in all 3 ST groups (-30% to -87%, Table 1). The introduction of PCV20 in a 2+1 or 3+1 regimen resulted in more bIPD cases from PCV7 and PCV13-non-ST3 STs. ST3 bIPD was maintained at current levels in a 2+1 regimen but decreased with PCV20 in a 3+1 regimen.

**Conclusion:**

Using published predicted VEs, implementation of PCV20 in a 2+1 or 3+1 regimen led to a substantial increase in bIPD incidence of PCV7 and PCV13 non-ST3 STs relative to currently used PCV13 2+1 regimen. PCV15 in a 2+1 regimen reduced bIPD incidence across all ST groups, with the largest impact on ST3. As higher-valent PCVs are introduced, the impact of their reduced ST-specific immune responses, and therefore lower predicted VE on pediatric PCV13 STs, should be weighed against the benefits of additional ST coverage.

^1^Ryman J, et al. Expert Rev Vaccines. 2024;23(1):60-68.

^2^Ryman J, et al. Expert Rev Vaccines. 2024;23(1):467-473.

**Disclosures:**

**Kevin Bakker, PhD**, Merck & Co., Inc.: Grant/Research Support|Merck & Co., Inc.: Stocks/Bonds (Public Company) **Rachel Oidtman, PhD**, Merck & Co., Inc.: Full time employee|Merck & Co., Inc.: Stocks/Bonds (Public Company) **Giulio Meleleo, PhD**, Merck & Co., Inc.: Vendor **Priscilla Velentgas, PhD**, Merck & Co., Inc., Rahway, NJ, USA: Grant/Research Support|Merck & Co., Inc., Rahway, NJ, USA: Stocks/Bonds (Public Company) **Kenneth Klinker, PharmD**, Merck & Co., Inc., Rahway, NJ, USA: Grant/Research Support|Merck & Co., Inc., Rahway, NJ, USA: Stocks/Bonds (Public Company) **Natalie Banniettis, MD**, Merck & Co., Inc., Rahway, NJ, USA: Grant/Research Support|Merck & Co., Inc., Rahway, NJ, USA: Stocks/Bonds (Public Company) **Kristen A. Feemster, MD, MPH, MSHPR, FAAP**, Merck & Co., Inc., Rahway, NJ, USA: Grant/Research Support|Merck & Co., Inc., Rahway, NJ, USA: Stocks/Bonds (Public Company) **Jessica Weaver, PhD, MPH**, Merck & Co. Inc.: Employment|Merck & Co. Inc.: Stocks/Bonds (Public Company)

